# Analyzing the Effects of Rapid and Natural Cooling Techniques on the Quality of Hand-Shaken Green Tea Beverages

**DOI:** 10.3390/foods13152322

**Published:** 2024-07-24

**Authors:** Yuan-Ke Chen, Tuzz-Ying Song, Chi-Yu Chang, Shiann-Cherng Sheu, Chih-Wei Chen

**Affiliations:** 1 Ph.D. Program of Biotechnology and Bioindustry, College of Biotechnology and Bioresources, Da-Yeh University, Changhua 515006, Taiwan; fsancohor@yahoo.com.tw; 2 Department of Medicinal Botanicals and Foods on Health Applications, Da-Yeh University, Changhua 515006, Taiwan; song77@mail.dyu.edu.tw (T.-Y.S.); feccy2@yahoo.com.tw (C.-Y.C.); 3 Bachelor Degree Program in Food Safety/Hygiene and Laboratory Science, Chang Jung Christian University, Tainan 711301, Taiwan; scschem@mail.cjcu.edu.tw

**Keywords:** hand-shaken green tea, rapid cooling, natural cooling, quality

## Abstract

This study compared the quality of hand-shaken green tea prepared through rapid and natural cooling methods. Cooling is crucial in preserving green tea’s flavor, aroma, and nutritional components. In the rapid cooling method, green tea is freshly brewed at an initial temperature of 95 °C for 25 min, and then rapidly cooled to 18 °C for 25 min. Conversely, the natural cooling method involves brewing tea at the same initial temperature and time, but allowing it to cool gradually to 30 °C over approximately 4–5 h at room temperature. This study’s findings indicate that the rapid cooling method produced green tea with a more vibrant color and improved clarity versus the natural cooling method. Sensory analysis revealed that the taste and aroma of the hand-shaken green tea prepared using rapid cooling were perceived to be more refreshing and invigorating. However, the natural cooling method preserved a higher level of chemical components, including individual catechin caffeine, total polyphenol, soluble solids, reducing sugar, and total tannins. The essential amino acid content of the rapidly and naturally cooled green tea infusions was 6.85 and 13.55 μg/mL, respectively. The γ-Aminobutyric acid (GABA) content was 439.82 and 457.31 μg/mL, respectively. This study’s findings suggest that rapid cooling during the preparation of hand-shaken green tea enhances its overall quality. The vibrant color, improved clarity, refreshing taste, and invigorating aroma make it a preferable choice for tea enthusiasts who seek an enhanced sensory experience and excellent quality.

## 1. Introduction

Following water, tea is the most widely consumed beverage worldwide. Its health benefits and medicinal use have been extensively investigated since early Chinese civilizations [1,2]. Three main types of tea have been classified based on their manufacturing process: green, black, and oolong tea. Green tea is a highly consumed beverage globally, particularly in Asia, due to its unique sensory characteristics and cultural significance [3].

Studies indicate that green tea provides numerous health benefits, including inhibiting the growth of harmful microbes and viruses, reducing the risk of cardiovascular diseases, and preventing the development of diabetes, obesity, cancer, and other ailments that involve oxidative stress [3,4,5,6]. Recent research indicates that green tea can help protect against coronaviruses, including COVID-19, by reducing inflammation-related complications [7,8]. The array of bioactive components in tea infusions, including polyphenols, caffeine, theanine, catechin, and free amino acids, provides such protection [3,4,5,9].

Two essential qualities of green tea infusion are its astringency and bitterness. Studies suggest that bitter substances often produce astringency, leaving a dry, rough sensation in the mouth [6,10,11]. Bitterness is typically a warning signal to avoid harmful substances; too much bitterness can lead to nausea [12]. However, some bitter and astringent components can enhance the taste of foods and beverages, such as green tea [11,13]. Moderate bitterness and astringency can enrich the flavor of green tea, and a sweet aftertaste often follows its bitterness or astringency [6,11]. Additionally, some bitter and astringent substances in tea have health benefits for humans [14].

The biological components of tea infusion impact human health and affect its quality. Certain compounds, such as tannins, catechins, and other phenolic compounds, can cause bitterness and astringency during tea infusion [11]. Alkaloids such as caffeine, theobromine, and theophylline are the primary bitter and astringent substances, with caffeine being the prime flavor-enhancing substance [8,10,11]. Green tea infusion typically comprises bitterness, umami, and a sweet aftertaste [6,15]. Astringency, a feeling on the tongue caused by the interaction of polyphenols (such as catechins, quercetin, and gallic acid) and salivary proteins, is a significant factor in the taste quality of green tea [6,15,16,17].

However, when controlling the concentration of bitter and astringent substances in green tea infusion, it is challenging to balance the flavor and health benefits while reducing discomfort from excessive bitterness and astringency [11].

The content of bioactive components in tea infusion depends primarily on the time and temperature at which the tea is brewed. Therefore, it is essential to pay attention to these factors. When brewing green tea, the brewing process plays a crucial role in the final quality of the infusion process. For traditional green tea, 3 g of tea should be steeped in 150 mL of boiling water for five minutes before it is ready to drink [18]. However, hand-shaken green tea requires 250 g of tea leaves and 18 L of boiling water. After steeping for 25 min, the tea leaves and the tea infusion should be separated and left to cool for approximately 4–5 h until the temperature reaches 27–30 °C. Therefore, the quality of hand-shaken green tea is determined by the speed at which it cools.

This study investigated the effects of two cooling methods, rapid and natural, on hand-shaken green tea infusion. We examined the levels of catechins, caffeine, total polyphenols, soluble solids, reducing sugar, total tannins, free amino acids, and GABA, and how they contribute to the bitterness and astringency of tea.

## 2. Materials and Methods

### 2.1. Chemicals

This study’s reagents and chromatographic grade standards, such as individual catechin (catechin (C), catechin gallate (CG), gallocatechin (GC), epicatechin (EC), epicatechin gallate (ECG), epigallocatechin (EGC), gallocatechin gallate (GCG), epigallocatechin gallate (EGCG)), caffeine, gallic acid, tannin, amino acid, and γ-Aminobutyric acid (GABA), were purchased from Sigma-Aldrich Chemical Co. (St. Louis, MO, USA). All chemical standards had a minimum purity of 98%. The green teas were obtained from the Tea Research and Extension Station (TRES) in Nantou, Taiwan.

### 2.2. Preparing the Green Tea Infusions

For this experiment, Taiwan Tea Experiment Station No. 12 (TTES No. 12, Jinxuan, Nantou, Taiwan) green tea, purchased from the Ruicheng Tea Factory in Nantou, Taiwan, was used. The tea infusions were separated into two groups for sensory evaluation and physicochemical analysis based on the use of either the natural or rapid cooling method.

**Figure 1:**
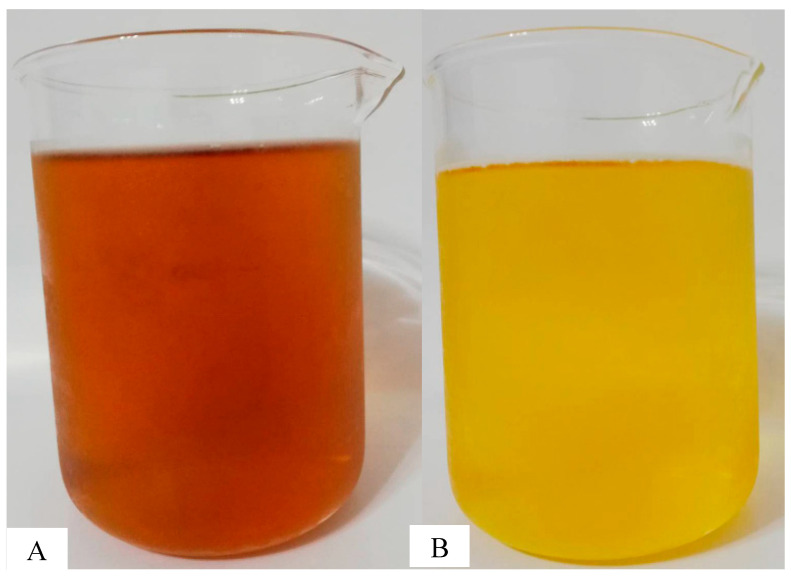
The green tea infusions prepared using two different cooling methods: (**A**) natural cooling (95 °C to 30 °C in 4–5 h, brewing time: 25 min) and (**B**) rapid cooling (95 °C to 18 °C in 25 min, brewing time: 25 min).

To naturally cool the tea, we brewed it with boiling water and allowed it to cool. We poured 18,000 mL of hot water at 95 °C into a tea barrel, then added 250 g of green tea leaves and stirred them well. We let the leaves brew for approximately 25 min before filtering them. The resulting tea infusion (Figure 1A) was cooled in a cold room to 30 °C, which took about 4–5 h, before packaging.

A NE-15 cooling machine (Chenguang Energy Technology Co., Ltd., Nantou, Taiwan) was used for rapid cooling. We followed the same process of boiling and pouring 18,000 mL of hot water at 95 °C into the tea barrel, adding 250 g of green tea leaves, and stirring well. It took about 25 min to brew and cool to 18 °C. We filtered out the tea infusion (see Figure 1B) with a filter and proceeded with subsequent packaging. The detailed temperature changes were as follows:

Natural Cooling Process:

- Initial temperature: 95 °C
- Brewing time: 25 min
- Final cooled temperature: 30 °C
- Cooling duration: Approximately 4–5 h

Rapid Cooling Process:

- Initial temperature: 95 °C
- Brewing time: 25 min
- Final cooled temperature: 18 °C
- Cooling duration: Approximately 25 min

### 2.3. Determining the Total Polyphenol Content

The measurements and modifications in the present study were based on Chiang et al. [5]. For each 0.5 mL of tea infusion, we added 7 mL of water, followed by 0.5 mL of Folin–Ciocalteu reagent, which was mixed for three minutes. Next, 2 mL of 20% Na_2_CO_3_ was added, and the samples were left in the dark at room temperature for two hours. The absorbance was read at 750 nm, and the calibration curve was determined using gallic acid solutions (R^2^ = 0.999). The phenolic content was expressed as mg gallic acid equivalents per gram of the extract (mg gallic acid/g).

### 2.4. Determining the Reducing Sugars and Soluble Solid Content

The measurements and modifications were performed following the method described by Chiang et al. [4]. Specifically, 2 mL of DNS reagent was added to each 1 mL of tea infusion and thoroughly mixed. The samples were then subjected to a five-minute boiling water bath and cooled to room temperature. We measured the absorbance at 540 nm and constructed a calibration curve using glucose concentrations ranging from 0.2 to 1.6 mg/mL (R^2^ = 0.995). The soluble solid content was measured using the AOAC method 22.024 [19]. The reducing sugars and soluble solids content were expressed in μg/mL.

### 2.5. Determining the Amino Acid and GABA Content

The green tea infusions were hydrolyzed using 10 mL of 6 M hydrogen chloride at 110 °C for 22 h under a nitrogen atmosphere to obtain the total amino acids. We used the Hitachi L-8900 Amino Acid Analyzer (Hitachi Co., Ltd., Tokyo, Japan) for the amino acid composition analysis. The amino acid and GABA content were expressed as μg/mL.

### 2.6. Determining the Total Tannin Content

Total tannin content was determined using the Folin–Ciocalteu method with modifications as per Farahmandfar et al. [20]. A 1 g sample was sonicated in 20 mL water for 30 min, followed by centrifugation. We mixed the supernatant (100 µL) with 1 mL of diluted Folin–Ciocalteu reagent and left it for five minutes. Next, 2 mL 7.5% sodium carbonate was added and incubated at 40 °C for 30 min. Absorbance was measured at 765 nm. A gallic acid standard curve (0–100 mg/L, R^2^ = 0.997) was used to calculate tannin content, expressed as mg gallic acid equivalents per gram (mg gallic acid/g) of the sample.

### 2.7. Determining the Individual Catechins and Caffeine Content

We analyzed the individual catechins and caffeine content using reversed-phase high-performance liquid chromatography (HPLC) with a UV-VIS spectrometric detector (Shimadzu RID-6A, Tokyo, Japan), as described by Chiang et al. [4]. A 5 μm Diamonsil C18 column (4.6 mm × 250 mm) was used, and the mobile phase comprised a mixture of water (0.1 mol/L H_3_PO_4_, 1.0 × 10^−4^ mol/L EDTA), acetonitrile, and ethyl acetate in a ratio of 85:12:3 (*v*/*v*). Elution was performed at a 1.0 mL/min solvent flow rate, and chromatograms were recorded at 280 nm. The individual catechins and caffeine content were expressed in μg/mL.

### 2.8. pH Value and Color Measurement

The pH value of the tea infusion was determined using a pH meter (model number: MP220, Swiss Merchant Mettler-Toledo Co., Ltd., Taiwan Branch (Kaohsiung, Taiwan). The colorimeter (model number: NE4000, Nippon Denshoku, Tokyo, Japan) was used according to the description of Lee et al. [21] to measure Hunter L, a, and b values to determine the colors of the two kinds of tea infusion. The comprehensive color difference was represented by ΔE using the following formula:

ΔE = [(ΔL)^2^ + (Δa)^2^ + (Δb)^2^]^1/2^

### 2.9. Sensory Analysis-Consumer Hedonic Tests

Concerning the sensory evaluation section of this study, we consulted the Research Ethics Committee (DYU-REC, 25 November 2023) at the outset. As a result, since the study did not involve sensitive data or risks to the participants, the experiment was not formally subjected to a research ethics review. To analyze the samples, this study recruited a panel of 60 semi-trained members (30 males and 30 females) aged 22 to 50 from the Department of Medicinal Botanicals and Foods on Health Applications at Da-Yeh University, Changhua, Taiwan. We conducted sensory evaluations using a 9-point hedonic scale (1 point: Dislike Extremely; 2 points: Dislike Very Much; 3 points: Dislike Moderately; 4 points: Dislike Slightly; 5 points: Neither Like nor Dislike; 6 points: Like Slightly; 7 points: Like Moderately; 8 points: Like Very Much; and 9 points: Like Extremely). The evaluations were conducted at a controlled temperature of 27 ± 2 °C. The overall acceptability of the samples was determined by calculating the average scores given for the attributes evaluated.

### 2.10. Statistical Analysis

The reported data were obtained through triplicate measurements of the samples. We performed statistical analysis using one-way analysis of variance (ANOVA) and *t*-test multiple comparisons in SAS 9.0 (SAS Institute, Cary, NC, USA). A significance level of *p* < 0.05 was considered statistically significant.

## 3. Results and Discussion

### 3.1. Effects of Different Brewing Cooling Methods on the pH Value, Soluble Solids, and Reducing Sugar Content of Tea Infusions

The pH values of the rapidly and naturally cooled green tea infusions were 6.36 and 7.44, respectively (Table 1).

The observed difference in pH between the two cooling methods can be attributed to several factors. During the rapid cooling process, the green tea infusion temperature drops quickly, potentially leading to increased proton release from certain compounds, thus lowering the pH. In contrast, the natural cooling process allows for a slower temperature decline, which may result in reduced proton release and a higher pH value.

The pH of green tea plays a significant role in determining its taste, flavor, and overall quality. Research indicates that variations in pH can influence the solubility and stability of tea components, such as catechins and amino acids, which are critical contributors to its sensory attributes and health benefits [22]. Moreover, pH affects the sensory perception of green tea, with lower pH values associated with a more pronounced and vibrant taste. Comparatively, higher pH values may result in a smoother, milder taste [23].

The difference in pH between the two cooling methods can have significant implications for green tea quality and consumer acceptance. Careful control of the cooling process to achieve the desired pH level is crucial for optimizing green tea production and meeting consumer preferences.

The cooling method used when preparing green tea infusions affects the levels of soluble solids and reducing sugars. Table 1 shows that this study’s green tea infusion cooled rapidly had a soluble solids content of 2714.58 μg/mL and a reducing sugar content of 609.93 mg/mL. In contrast, the natural cooling tea infusion had a soluble solids content of 3476.94 μg/mL and a reducing sugar content of 799.84 mg/mL. The cooling rate and duration caused these differences. When cooled rapidly, the soluble solids may be better preserved due to the sudden temperature drop, inhibiting the breakdown of certain compounds. In contrast, a slower cooling rate during natural cooling may allow for more chemical reactions, leading to a higher release of soluble solids and reducing sugars in the tea infusion. Soluble solids content is a vital indicator of the dissolved compounds in green tea that contribute to its taste, flavor, and overall quality. These compounds include catechins, amino acids, and other flavor components [22]. Similarly, reducing sugar enhances a tea’s sweetness and mouthfeel.

The difference in soluble solids and reducing sugar contents between the two cooling methods can affect green tea’s perceived taste and flavor profile. Consumers may have varying preferences for sweetness levels and mouthfeel, making the control of cooling conditions crucial in meeting these preferences.

The soluble solid and reducing sugar contents are crucial in green tea infusions’ sensory qualities and overall quality. Soluble solid content affects a tea’s body and mouthfeel, while the reducing sugars add to the sweetness and flavor profile. Higher soluble solid content produces a fuller-bodied tea with a richer taste, while increased reducing sugar content enhances a tea’s sweetness and overall sensory perception. These attributes benefit those seeking a more flavorful and enjoyable tea experience.

Furthermore, green tea’s soluble solids and reducing sugars interact with other components, such as catechins and amino acids, affecting their stability and bioavailability. These interactions influence the sensory attributes, antioxidant activity, and potential health benefits of green tea consumption [4,5].

### 3.2. Effects of Different Brewing Cooling Methods on the Total Tannins, Total Polyphenol, and Caffeine Content of Tea Infusions

The cooling method used in preparing green tea infusions significantly impacts the total tannin, total polyphenol, and caffeine content. The naturally cooled green tea infusion in the present study had higher levels of total tannins (1157.65 mg gallic acid/g), total polyphenol (1449.21 mg gallic acid/g), and caffeine (398.49 μg/mL) compared to the rapidly cooled infusion (785.47 mg gallic acid/g, 972.85 mg gallic acid/g, and 263.41 μg/mL, respectively) (Table 1). The longer steeping time during natural cooling resulted in a more efficient extraction of tannins, polyphenols, and caffeine from the tea leaves, leading to higher concentrations in the infusion. The differences in total tannin and polyphenol content can be attributed to the cooling process and its effect on the extraction efficiency of these compounds from the tea leaves. Natural cooling allows for a longer steeping time, which promotes greater extraction of tannins and polyphenols. Tannins and polyphenols contribute to the astringency and bitterness of tea, and longer steeping times result in higher levels of these compounds. In a previous study [4], the caffeine and polyphenol content was shown to increase significantly with longer steeping times in green tea infusions.

The higher caffeine content in the natural cooling green tea infusion may be attributed to the prolonged exposure to caffeine during natural cooling. Caffeine is a naturally occurring compound in tea extracted during the steeping process. The longer steeping time with natural cooling allows for more efficient extraction of caffeine from the tea leaves, resulting in higher levels in the infusion.

The total tannins, total polyphenols, and caffeine are essential parameters that can impact the sensory qualities and overall quality of green tea infusions. Tannins and polyphenols contribute to a tea’s astringency, bitterness, and antioxidant activity. Higher levels of these compounds may result in a more pronounced astringent and bitter taste. Caffeine content contributes to tea’s stimulant effects and can influence the overall perception of its sensory attributes. Moreover, tannins, polyphenols, and caffeine are bioactive compounds in green tea associated with various health benefits. These compounds possess antioxidative, anti-inflammatory, and potential anticancer properties. Higher tannins, polyphenols, and caffeine in a naturally cooled green tea infusion may enhance its potential health benefits. Research suggests that the polyphenol content and antioxidant activity of green tea infusions are positively correlated [4,5].

The present study’s ratio of polyphenols to amino acids in green tea infusions showed a significant difference between the rapidly and naturally cooled methods (Table 1). The rapidly cooled green tea infusion had a ratio of polyphenols to amino acids of 12.17, while the naturally cooled green tea infusion had a higher ratio of 20.02.

The ratio of polyphenols to amino acids is an essential indicator of the balance between these two groups of compounds in green tea. Polyphenols, such as catechins, are abundant in green tea and contribute to its antioxidative, health-promoting properties. On the other hand, amino acids play a vital role in the taste and flavor of tea, particularly in providing its umami and sweet notes [4,5,24]. The ratio of polyphenols to amino acids in green tea infusions significantly impacts their taste, flavor, and overall quality. A higher ratio may lead to a more robust and astringent taste due to the increased presence of polyphenols. A lower ratio may result in a milder flavor with more pronounced umami and sweetness from the amino acids [15].

### 3.3. Effect of Different Brewing Cooling Methods on Individual Catechin Content of Tea Infusions

The amounts of individual catechins in green tea infusions made using the rapid and natural cooling methods are shown in Table 2. The individual catechin contents in the green tea infusion made using the rapid cooling process were as follows: gallocatechin (GC) 33.62 μg/mL, epigallocatechin (EGC) 126.01 μg/mL, catechin (C) 254.64 μg/mL, epicatechin (EC) 23.98 μg/mL, epigallocatechin gallate (EGCG) 5.28 μg/mL, gallocatechin gallate (GCG) 83.61 μg/mL, epicatechin gallate (ECG) 46.54 μg/mL, and catechin gallate (CG) 52.15 μg/mL. The individual catechin contents in the naturally cooled green tea infusion were as follows: GC 85.65 μg/mL, EGC 91.16 μg/mL, C 246.32 μg/mL, EC 127.36 μg/mL, EGCG 25.72 μg/mL, GCG 100.53 μg/mL, ECG 55.50 μg/mL, and CG 74.21 μg/mL.

The differences in individual catechin contents can be attributed to the cooling process and its impact on extraction and transformation. Natural cooling, with a longer steeping time, allows for more efficient extraction of catechins from the tea leaves. Additionally, catechins can undergo oxidation and conversion into other forms during natural cooling. Studies indicate that the longer steeping time and exposure to oxygen during natural cooling can lead to the conversion of catechins into more complex, polymerized forms, such as theaflavins (TF) and thearubigins (TA) [5,6,25].

Different catechin profiles can significantly influence the sensory qualities and overall quality of green tea infusions. For example, EGCG is one of green tea’s most abundant, biologically active catechins, known for its antioxidative and potential health-promoting effects [26]. Higher levels of EGCG, as observed in the natural cooling infusion, may contribute to increased antioxidant activity and potential health benefits.

Furthermore, individual catechins contribute to green tea’s flavor profile and astringency. Catechins such as EGC, C, and EC are associated with bitterness and astringency [5,11,27]. Higher levels of these catechins, as seen in the natural cooling infusion, may produce a more pronounced bitter and astringent taste.

### 3.4. Effect of Different Brewing Cooling Methods on Amino Acids, EAA, and GABA Contents of Tea Infusions

The amino acid content of the green tea infusions in the present study showed significant differences between the rapid and natural cooling methods (Table 3). The rapid cooling green tea infusion had a total amino acid content of 79.96 μg/mL, while the natural cooling green tea infusion had a slightly lower content of 72.40 μg/mL. Among the essential amino acids (EAA), the naturally cooled green tea infusion exhibited higher levels of glycine (Gly), alanine (Ala), arginine (Arg), threonine (Thr), valine (Val), isoleucine (Ile), leucine (Leu), and phenylalanine (Phe) than its rapidly cooled counterpart. In contrast, the rapidly cooled green tea infusion had higher glutamic acid (Glu), aspartic acid (Asp), tyrosine (Tyr), tryptophan (Trp), and lysine (Lys) levels. However, the cysteine (Cys), serine (Ser), proline (Pro), and methionine (Met) levels did not differ significantly between the two cooling methods. Additionally, no significant difference was observed in GABA content, with values of 439.82 and 457.31 μg/mL for the rapidly and naturally cooled infusions, respectively.

Variations in amino acid content can significantly impact the taste, flavor, and overall quality of green tea infusions. Amino acids contribute to a tea’s umami, sweetness, bitterness, and astringency [28,29]. Therefore, the balance and levels of different amino acids play a crucial role in determining the sensory attributes of tea. Interestingly, the two cooling methods showed no significant difference in this study’s GABA (gamma-aminobutyric acid) content. GABA is known for its calming and relaxing effects and contributes to the unique sensory profile of green tea [29].

Notably, the amino acids Cys, Ser, Pro, and Met showed no significant differences between the two cooling methods. These amino acids appear to have stable levels in green tea infusions and are less affected by the cooling process.

### 3.5. Effect of Different Brewing Cooling Methods on the Color of Tea Infusions

**Figure 2:**
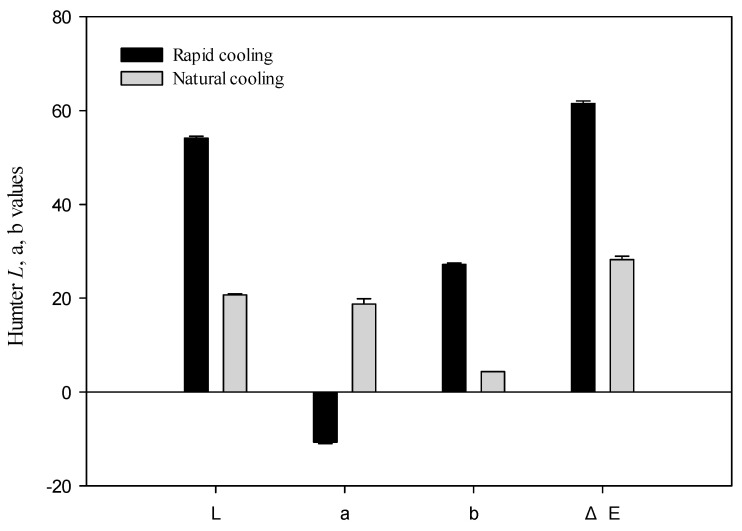
Chromatic aberration analysis of rapid and natural cooling green tea infusion. Results from three separate experiments are expressed as mean ± SD.

Two different cooling methods for green tea infusion are shown in Figure 1. The color of the green tea infusions exhibited significant differences between the rapid and natural cooling methods we tested during this study (Figure 2). The L-values, representing lightness, were higher in the rapid cooling green tea infusion (L = 54.13) than in the natural cooling green tea infusion (L = 20.68). The a-values, indicating redness-greenness, were lower in the rapid cooling green tea infusion (a = −10.71) than in the natural cooling green tea infusion (a = 18.73). The b-values, representing yellowness-blueness, were higher in the rapid cooling green tea infusion (b = 27.19) than in the natural cooling infusion (b = 4.53). Moreover, the ΔE-values, indicating the overall color difference, were more significant in the rapid cooling infusion (ΔE = 61.51) than in the natural cooling infusion (ΔE = 28.25).

The color of green tea is influenced by various factors, including the content of chlorophyll, flavonoids, and other pigments, as well as by oxidation and degradation processes. Steeping conditions, such as temperature and duration, can affect the extraction and transformation of these pigments, resulting in color variations.

Therefore, the color of green tea is a critical quality attribute that can affect consumer perception, as it is often associated with freshness, taste, and overall quality. Lighter and yellow-green colors are generally preferred, indicating higher quality, better appearance, and a tea with improved mouthfeel [15].

### 3.6. Effect of Brewing Temperature and Duration on the Sensory Analysis-Consumer Hedonic Tests

**Figure 3:**
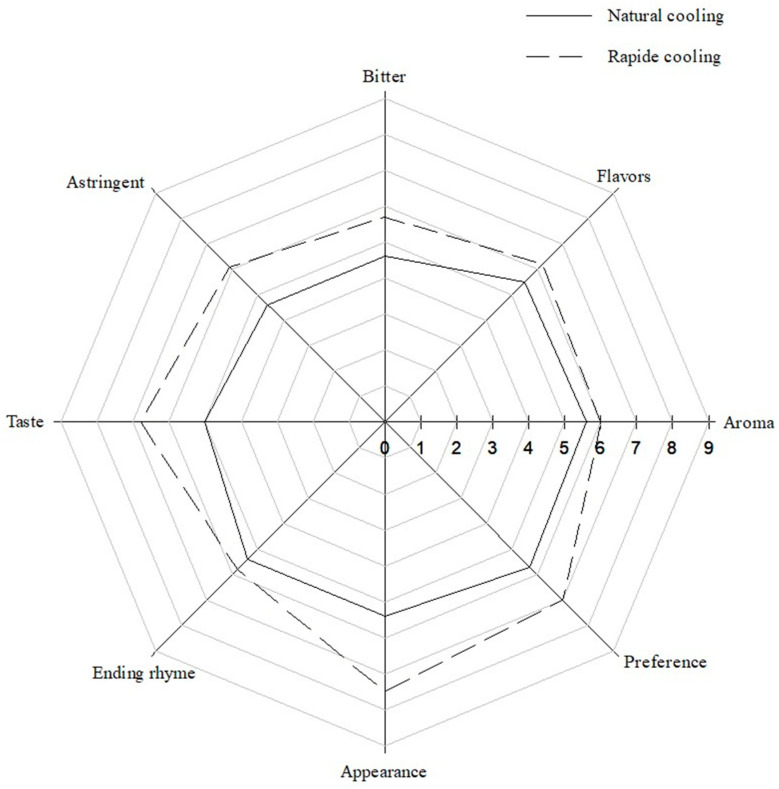
Radar chart of consumers’ acceptance of tea sensory evaluation under rapid and natural cooling.

According to the consumer hedonic tests in Figure 3, the sensory analysis indicates that the green tea infusion with rapid cooling had a better aroma, flavor, taste, aftertaste, appearance, and higher overall acceptability than the natural cooling green tea infusion. The differences between the two cooling methods were particularly significant in terms of taste, appearance, and preference.

Green tea’s taste and appearance are crucial factors influencing consumer preferences. This study’s naturally cooled green tea had significantly higher bitter and astringent tastes than the rapidly cooled green tea. This finding could be attributed to the longer steeping time during the natural cooling process, which increased the extraction of bitter-tasting catechins and astringent compounds from the tea leaves.

Rapid cooling preserves green tea’s delicate and fresh characteristics better than natural cooling. A faster cooling rate helps retain volatile aroma compounds essential for the overall sensory experience. The higher aroma, flavor, and aftertaste scores for the rapid cooling infusion might be due to the better preservation of these volatile compounds. Figure 3 indicates that consumers preferred the rapidly cooled green tea infusions regarding color, aroma, and taste.

## 4. Conclusions

This study comparing rapid and natural cooling methods for green tea revealed significant differences in pH, soluble solids, reducing sugars, total tannins, polyphenols, caffeine, catechins, amino acids, and color between the two methods. These differences are crucial for determining the flavor, aroma, and overall sensory experience of tea. Rapid cooling resulted in a lower pH (6.36) compared to natural cooling (7.44), likely due to different rates of chemical reactions. Additionally, rapid cooling yielded lower soluble solids and reducing sugars, indicating decreased solubility and sugar extraction. In contrast, natural cooling produced higher amounts of total tannins, polyphenols, and caffeine, enhancing the extraction of these compounds.

The catechin profile varied between the two methods, influencing the flavor and aroma of the produced teas. Natural cooling increased essential amino acids and those associated with pleasant tastes and aromas. Color differences were also noted, with rapid cooling resulting in a lighter, greener, and yellower tea, while natural cooling produced a darker, redder infusion. Sensory analysis favored the rapid cooling method, with higher scores in aroma, flavor, taste, aftertaste, and appearance, while natural cooling led to more bitter and astringent tastes, potentially affecting consumer perception.

These findings underscore the importance of the cooling method in green tea production. It impacts consumer acceptability and perception of tea quality. Tea producers and researchers should consider the effects of the cooling process in order to optimize tea quality and meet consumer preferences and market demands.

## Figures and Tables

**Table 1:** The pH value, soluble solids, reducing sugar, total tannins, total polyphenol, and caffeine contents of rapid cooling and natural cooling green tea infusions.

Items	Green Tea Infusion	
	Rapid Cooling Green Tea	Natural Cooling Green Tea
pH value	6.36 ± 0.15 ^B,^*	7.44 ± 0.11 ^A^
Soluble solids (μg/mL)	2714.58 ± 143.74 ^B^	3476.94 ± 188.39 ^A^
Reducing sugar (μg/mL)	609.93 ± 73.41 ^B^	799.84 ± 46.83 ^A^
Total tannins (mg gallic acid/g)	785.47 ± 36.72 ^B^	1157.65 ± 61.88 ^A^
Total polyphenol (mg gallic acid/g)	972.85 ± 53.82 ^B^	1449.21 ± 84.51 ^A^
Caffeine (μg/mL)	263.41 ± 26.75 ^B^	398.49 ± 19.63 ^A^

* Results from three separate experiments are expressed as mean ± SD. A,B: Data with identical letters in the same row are not significantly different (*p* < 0.05).

**Table 2:** Individual catechin content of rapid cooling and natural cooling green tea infusions.

Type of Individual Catechins (μg/mL)	Rapid Cooling Green Tea	Natural Cooling Green Tea
Gallocatechin (GC)	33.62 ± 1.54 ^B,^*	85.65 ± 1.39 ^A^
Epigallocatechin (EGC)	126.01 ± 5.54 ^A^	91.16 ± 1.46 ^B^
Catechin (C)	254.64 ± 12.71 ^A^	246.32 ± 2.78 ^A^
Epicatechin (EC)	23.98 ± 1.62 ^B^	127.36 ± 1.86 ^A^
Epigallocatechin gallate (EGCG)	5.28 ± 0.74 ^B^	25.72 ± 1.01 ^A^
Gallocatechin gallate (GCG)	83.61 ± 3.43 ^B^	100.53 ± 0.24 ^A^
Epicatechin gallate (ECG)	46.54 ± 2.69 ^B^	55.50 ± 0.83 ^A^
Catechin gallate (CG)	52.15 ± 1.98 ^B^	74.21 ± 0.70 ^A^

* Results from three separate experiments are expressed as mean ± SD. A,B: Data with identical letters in the same row are not significantly different (*p* < 0.05).

**Table 3:** Amino acid and GABA content of the rapid and natural cooling green tea infusions.

Free Amino Acid (μg/mL)	Green Tea Infusion	
	Rapid Cooling	Natural Cooling
Glu	21.68 ± 0.27 ^A,^*	17.12 ± 0.35 ^B^
Asp	24.0 ± 0.52 ^A^	20.05 ± 0.28 ^B^
Cys	4.10 ± 0.13 ^A^	4.35 ± 0.04 ^A^
Ser	2.13 ± 0.11 ^A^	2.47 ± 0.03 ^A^
Gly	0.65 ± 0.01 ^B^	2.12 ± 0.02 ^A^
His	ND **	ND
Ala	1.18 ± 0.02 ^B^	1.93 ± 0.05 ^A^
Arg	1.37 ± 0.06 ^B^	2.65 ± 0.05 ^A^
Thr	0.25 ± 0.00 ^B^	0.78 ± 0.02 ^A^
Pro	0.18 ± 0.01 ^A^	0.17 ± 0.00 ^A^
Tyr	11.82 ± 0.13 ^A^	7.99 ± 0.06 ^B^
Val	0.46 ± 0.01 ^B^	1.68 ± 0.04 ^A^
Met	3.43 ± 0.07 ^A^	3.61 ± 0.02 ^A^
Ile	0.32 ± 0.01 ^B^	0.55 ± 0.02 ^A^
Lue	0.51 ± 0.01 ^B^	4.33 ± 0.05 ^A^
Phe	0.26 ± 0.00 ^B^	1.46 ± 0.03 ^A^
Trp	1.45 ± 0.05 ^A^	0.98 ± 0.01 ^B^
Lys	0.26 ± 0.01 ^A^	0.16 ± 0.01 ^B^
EAA ***	6.85 ± 0.13 ^B^	13.55 ± 0.19 ^A^
Total	79.96 ± 0.38 ^A^	72.40 ± 0.34 ^B^
GABA (μg/mL)	439.82 ± 15.93 ^A^	457.31 ± 11.58 ^A^

* Results from three separate experiments are expressed as mean ± SD. A,B: Data with identical letters in the same row are not significantly different (*p* < 0.05). ** ND: not detected. *** EAA: essential amino acid.

## Data Availability

The original contributions presented in the study are included in the article, further inquiries can be directed to the corresponding author.
